# Aortic Valve Neocuspidization: Promise Without Proof for the Young Patient

**DOI:** 10.1016/j.jacadv.2026.102716

**Published:** 2026-05-27

**Authors:** Jacques Tomasi

**Affiliations:** Department of Cardio-Thoracic Surgery, University Hospital of Rennes, Rennes, France

We read with interest the paper by Ozaki et al^1^ reporting midterm outcomes of aortic valve neocuspidization (AVNeo) in 1,196 patients, with follow-up extending to 12 years. Although the authors are to be congratulated for this extensive clinical experience, several concerns arise, especially regarding application in younger patients.

Despite the procedure being introduced in 2007, the longest reported follow-up remains capped at 12 years, with only 5% of patients followed beyond 10.5 years.^2^ This limitation restricts evaluation of true long-term durability, which is essential when considering AVNeo in younger individuals with life expectancy of several decades.

Furthermore, progressive aortic regurgitation has been reported. The incidence of moderate or greater regurgitation rose from 0.3% at 6 months to 6.6% at 10 years.^1^ Structural valve degeneration requiring reintervention reached ∼9% at 10 years in patients under 55, vs ∼1% in those over 80.^3^^,^^4^ These trends mirror the well-known limitations of biological valves in younger patients.

Infective endocarditis (IE) also remains a concern. In the Ozaki series, nearly half of all reoperations were triggered by IE, including in previously healthy valves.^1^^,^^5^ Although AVNeo avoids prosthetic material, autologous pericardium does not appear immune to infection-related complications.

Importantly, the procedure is not currently included in the 2025 European association of Cardiology, EACTS is European Association for CardioThoracic Surgery guidelines on valvular heart disease, which emphasize established strategies such as mechanical or bioprosthetic aortic valve replacement and the Ross procedure.^4^ The absence of AVNeo from international recommendations underlines the need for further validation.

Comparatively, the Ross procedure offers compelling long-term durability. Studies report >85% freedom from reintervention at 12 years, with 25-year survival reaching 76%, comparable to the general population. IE rates are notably low (0.2% per patient-year), and autograft failure remains rare.^5^

In conclusion, although AVNeo provides promising early hemodynamic outcomes, its limited follow-up, rising rates of valve insufficiency and IE, and absence from current guidelines argue for caution. Until long-term outcomes exceeding 15 to 20 years are available, AVNeo should not replace validated approaches such as the Ross procedure or mechanical aortic valve replacement in young patients.
